# Hydrochlorothiazide and chlorthalidone use and glaucoma risk: pharmacovigilance analysis and nationwide cohort study

**DOI:** 10.3389/fphar.2026.1768133

**Published:** 2026-03-10

**Authors:** Jooyoung Yoon, Jiyeong Kim, Ko Eun Kim, Jee-Eun Chung, Seong Joon Ahn

**Affiliations:** 1 Department of Ophthalmology, Hanyang University Guri Hospital, Guri, Gyeonggi-do, Republic of Korea; 2 Department of Pre-Medicine, College of Medicine, and Biostatistics Laboratory, Medical Research Collaborating Center, Hanyang University, Seoul, Republic of Korea; 3 Department of Ophthalmology, Asan Medical Center, University of Ulsan College of Medicine, Seoul, Republic of Korea; 4 College of Pharmacy and Institute of Pharmaceutical Science and Technology, Hanyang University, Ansan, Republic of Korea; 5 Department of Ophthalmology, Hanyang University Seoul Hospital, Hanyang University College of Medicine, Seoul, Republic of Korea

**Keywords:** adverse event reporting system, chlorthalidone, diuretics, glaucoma, hydrochlorothiazide, pharmacovigilance, thiazide

## Abstract

Hydrochlorothiazide and chlorthalidone are widely used thiazide diuretics, and pharmacovigilance reports have suggested a possible link between their use and glaucoma. Therefore, this study aims to evaluate whether the use of hydrochlorothiazide or chlorthalidone is associated with an increased risk of glaucoma in a nationwide population-based claims cohort to clarify the true risk and guide clinical practice. Pharmacovigilance disproportionality analyses of the Food and Drug Administration Adverse Event Reporting System (FAERS) reports were conducted to identify candidates for glaucoma-related diuretics. In the nationwide Korean Health Insurance Review and Assessment (HIRA) cohort of new users of hydrochlorothiazide or chlorthalidone, incidence of overall and subtype-specific glaucoma was assessed, and pre- and post-exposure incidence rate ratios (IRRs) were calculated. Multivariate Cox proportional hazards models were used to estimate hazard ratios (HRs) for clinical risk factors. Hydrochlorothiazide and chlorthalidone demonstrated positive disproportionality signals for glaucoma (ROR, 3.34; 95% confidence interval [CI], 2.85–3.91 and 11.37; 95% CI, 7.67–16.86, respectively). In the HIRA cohort of new users of hydrochlorothiazide (n = 250,296) or chlorthalidone (n = 10,005) from January 2019 to December 2020, the cumulative incidence of glaucoma/ocular hypertension rose gradually for both drugs, reaching approximately 2%–3% by study end. Pre- versus post-exposure IRRs for overall glaucomatous conditions were 1.02 (95% CI 0.98–1.06) for hydrochlorothiazide and 1.03 (95% CI 0.84–1.26) for chlorthalidone; IRRs for ocular hypertension were 1.02 (95% CI 0.97–1.07) and 1.09 (95% CI 0.87–1.37), respectively. Multivariate analyses found no significant association between the drugs and overall glaucomatous conditions (adjusted HR 1.01; 95% CI 0.86–1.19). In dose-stratified analyses, hydrochlorothiazide showed a modest increase in glaucoma risk in the highest cumulative exposure group (for overall glaucomatous conditions, HR 1.21; 95% CI 1.12–1.31; log-rank *P* < 0.01), whereas chlorthalidone showed no consistent dose–response trends. Although the FAERS pharmacovigilance suggested a potential glaucoma risk with hydrochlorothiazide and chlorthalidone, population-based validation found no significant association. Pharmacovigilance signals should be confirmed with population-based data to identify true drug–glaucoma associations.

## 1 Introduction

Glaucoma is a chronic, progressive disease causing irreversible visual field loss, making early detection critical. Besides aging, medications and systemic conditions, such as hypertension (Bae et al., 2014), diabetes (Zhou et al., 2014), and kidney disease (Cho et al., 2021), are potential risk factors. With an aging population and growing long-term pharmacotherapy use, understanding medication effects on intraocular pressure (IOP) and glaucoma is increasingly important.

Postmarketing safety surveillance systems, such as the United States Food and Drug Administration Adverse Event Reporting System (FAERS), monitor potential drug-related adverse events (Fang et al., 2014; Beninger, 2018). Recent FAERS analyses suggest associations between glaucoma and several drug classes, including adrenergic (Aftab et al., 2024; Wu et al., 2025), anticholinergic (Wu et al., 2025), sulfonamide (Aftab et al., 2024; Wu et al., 2025), serotonergic (Aftab et al., 2024), and psychotropic (Wu et al., 2025) agents. However, further validation is needed to confirm causal relationships (Beninger, 2018; Fusaroli et al., 2024). To address this, we developed a validation framework using the Korean Health Insurance Review and Assessment Service (HIRA) database to evaluate systemic drugs potentially associated with maculopathy, as identified through FAERS analyses, in a real-world population (Kim et al., 2025a). This framework integrates pharmacovigilance signal detection with population-based evidence, to better understand the ocular safety of systemic drugs.

Diuretics are widely prescribed for conditions including hypertension, renal or hepatic disease, heart failure, and edema; however, their relationship with glaucoma remains unclear. Previous findings suggested retinal ganglion cell loss is associated with diuretic use in a multi-ethnic Asian cohort (Chong et al., 2021), while analyses such as the DIN-LINK (UK) case–control study (Owen et al., 2010) or FAERS-based analyses (Wu et al., 2025) indicated a higher risk of glaucoma with thiazide use. In contrast, large population-based studies (Gutenberg (Laspas et al., 2024), Rotterdam (Muskens et al., 2007), and European Glaucoma Prevention Study (European Glaucoma Prevention Study et al., 2007)) found no significant associations between diuretic use and changes in IOP or incident open-angle glaucoma. For angle-closure glaucoma, case reports of hydrochlorothiazide (Geanon and Perkins, 1995; Lee et al., 2007; Chen et al., 2014; Wu et al., 2023; Premkumar et al., 2025), chlorthalidone (Singer et al., 2015; Durai et al., 2016), indapamide (Vegh et al., 2013; Pedrosa et al., 2018), and furosemide (Boundaoui and Woodruff, 2016), as well as the FAERS pharmacovigilance study (Aftab et al., 2024), suggest possible associations; yet a nested case–control analysis using the PharMetrics Plus database (United States) showed no increased risk of glaucoma with current diuretic use (Qiao et al., 2024).

Accordingly, we applied a two-step approach: first, we conducted FAERS disproportionality analyses to identify diuretic candidates potentially associated with glaucoma; second, using data from the HIRA cohort, we evaluated the association between diuretic use and glaucoma incidence in a real-world population.

## 2 Materials and methods

This study employed a two-phase design, integrating pharmacovigilance signal detection using the FAERS database with population-based analyses from the HIRA database to assess systemic drugs associated with glaucoma. This study was approved by the Institutional Review Board (IRB) of Hanyang University Hospital (IRB File No. 2025-03-016) and conducted following the principles of the Declaration of Helsinki. Informed consent was waived due to its retrospective nature and use of anonymized FAERS and HIRA data. Our analyses using FAERS and HIRA data ensured adherence to the Reporting of a Disproportionality Analysis for Drug Safety Signal Detection using Individual Case Safety Reports in Pharmacovigilance recommendations (Fusaroli et al., 2024), and the Strengthening the Reporting of Observational Studies in Epidemiology guidelines for observational research (von Elm et al., 2007).

### 2.1 Identification of candidate diuretics using the Food and Drug Administration Adverse Event Reporting System database

We queried the FAERS database for spontaneous reports of glaucoma-related terms associated with diuretics. Reports submitted between July 2014 and December 2024 were retrieved using MedDRA version 26.0 preferred terms to ensure comprehensive capture of relevant adverse events (Supplementary Table S1). After deduplication and restriction of reports where the drug was the primary suspect, disproportionality analyses were performed by calculating reporting odds ratios (RORs) with 95% confidence intervals (CIs) for representative agents across diuretic classes (e.g., hydrochlorothiazide, chlorthalidone, thiazide-like diuretics, loop diuretics, and potassium-sparing diuretics). Disproportionality signals were defined as ROR ≥2.0 with the lower bound of the 95% confidence interval >1.0, information component (IC) with IC025 (lower 95% credibility bound) >0, empirical bayes geometric mean (EBGM) with EBGM05 (5th percentile lower bound) >2.0 (Sakaeda et al., 2013), alongside ≥10 reports per drug-event pair. Diuretics were ranked by number of glaucoma-related reports (Table 1), and those meeting the prespecified threshold were selected for further claims-based evaluation to prioritize higher-plausibility candidates and reduce false-positive signals arising from chance findings and reporting-related artifacts (Slattery et al., 2013).

**TABLE 1:** Diuretics with the highest number of reported glaucoma adverse events (December 2024).

Rank	Medication	No. Of reports	ROR (95% CI)	IC (IC025)	EBGM (EBGM05)
1	Hydrochlorothiazide	155	3.34 (2.85–3.91)	1.73 (1.48)	3.32 (2.91)
2	Furosemide	35	0.99 (0.71–1.38)	−0.01	0.99 (0.75)
3	Chlorthalidone	25	11.37 (7.67–16.86)	3.50 (2.36)	11.30 (8.13)
4	Spironolactone	13	1.16 (0.67–2.00)	0.22 (0.12)	1.16 (0.74)
5	Triamterene	4	3.12 (1.17–8.31)	1.64 (0.61)	3.11 (1.37)
6	Indapamide	3	1.08 (0.35–3.36)	0.11 (0.04)	1.08 (0.42)
7	Torsemide	2	0.52 (0.13–2.08)	−0.94	0.52 (0.16)
8	Bumetanide	0	NA	NA	NA
9	Amiloride	0	NA	NA	NA

ROR, reporting odds ratio; CI, confidence interval; IC, information component; IC025, lower 95% credibility interval bound (2.5th percentile) of the information component; EBGM, empirical bayes geometric mean; EBGM05, 5th percentile lower bound of the empirical bayes geometric mean; NA, not applicable (no reports).

### 2.2 Cohort study evaluating glaucoma risk using the Korean Health Insurance Review and Assessment Service database

**FIGURE 1:**
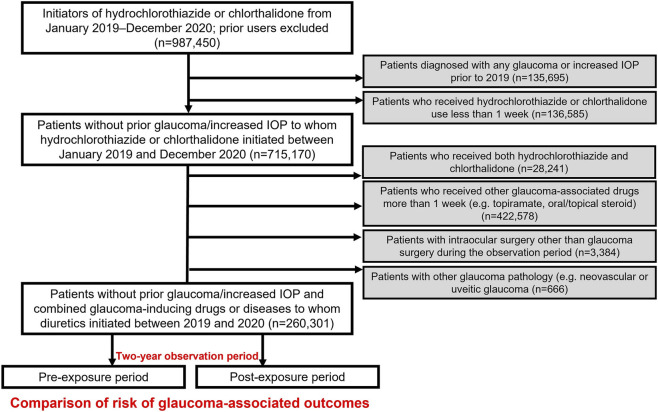
Schematic diagram of the study design using the Korean Health Insurance Review and Assessment Service (HIRA) database to evaluate glaucoma risk associated with candidate drugs.

For the population-based analyses, we employed the HIRA database, which captures healthcare claims for approximately 98% of the South Korean population (nearly 50 million individuals) through the country’s universal health insurance system (Kim et al., 2017). Within HIRA, we identified patients prescribed one of the candidate drugs identified through the FAERS analyses—hydrochlorothiazide or chlorthalidone—between 1 January 2018, and 31 December 2020. To include new users, those with any prior prescription during the 1-year period preceding the observation window were excluded, thereby improving causal interpretability by anchoring follow-up at the first prescription (index date) and reducing immortal time bias and carryover effects from prior exposure. The index date was defined as the date of the first prescription of hydrochlorothiazide or chlorthalidone between 1 January 2019 and 31 December 2020, following the 1-year washout period (January 2018 to December 2018). Follow-up began on the index date and continued regardless of treatment discontinuation; participants were censored at the earliest of (i) occurrence of a glaucoma outcome, (ii) the end of the study period (31 December 2020), or (iii) loss of eligibility in HIRA (predominantly due to death). To establish a clear association between drug exposure and glaucoma, patients with a history of glaucoma or ocular hypertension before 2019 were excluded, as were those with <1 week of cumulative exposure to a candidate drug, to ensure a minimum exposure requirement. Additional exclusions were (i) prescription of both candidate drugs, including switching between hydrochlorothiazide and chlorthalidone, to avoid exposure misclassification, (ii) receipt of medications known to induce glaucoma (e.g., topiramate, oral or topical corticosteroids), (iii) intraocular surgery history, other than glaucoma surgery, or (iv) diagnosis of other glaucoma pathologies, such as neovascular or uveitic glaucoma. For glaucoma diagnosis, a claims-based outcome definition was applied, defining cases as patients with ≥2 outpatient claims bearing the same glaucoma subtype diagnosis recorded by an ophthalmologist, in accordance with previous studies (Choi et al., 2018). Supplementary Table S2 provides the diagnostic codes used in HIRA to identify glaucoma, and Figure 1 presents a schematic overview of the HIRA analysis.

### 2.3 Statistical analysis

The incidence rates of glaucoma, including overall glaucomatous conditions, angle-closure glaucoma, open-angle glaucoma, and ocular hypertension, before and after exposure, were expressed as events per person-year and evaluated for each drug. To estimate relative risk, incidence rate ratios (IRRs) were calculated by comparing post-exposure versus pre-exposure incidence, as in prior self-controlled designs (Kim et al., 2025a; Kim et al., 2025b), thereby evaluating changes in glaucoma risk after drug initiation while controlling for fixed individual-level characteristics. Time-to-event outcomes were evaluated using Kaplan–Meier survival analysis to estimate cumulative risk. For each glaucoma type, cumulative incidences at 6 months, 1 year, and at study completion were calculated, and age- and sex-adjusted incidences were computed. Multivariate Cox proportional hazards models were used to identify risk factors associated with overall glaucomatous conditions and its subtypes, including actual glaucoma, angle-closure glaucoma, open-angle glaucoma, and ocular hypertension. The risk of glaucoma was evaluated by modeling use of hydrochlorothiazide versus chlorthalidone, with the alternate drug as the reference group. Because the analytic cohort was restricted to users of either hydrochlorothiazide or chlorthalidone, “non-use” of one agent corresponds to use of the other. Direct comparisons with untreated individuals would be prone to substantial confounding due to large baseline differences between treated and untreated populations, which this design partly alleviates. The multivariate Cox proportional hazards models were adjusted for age, sex, comorbidities (including hypertension, diabetes mellitus, kidney disease, and liver disease), and continuous variables on the drug use, including duration of use and daily dose. All variables were assessed at baseline (at the index date). Dose–response relationships were assessed using Cox proportional hazards models, with the lowest cumulative dose group serving as the reference. Hazard ratios (HRs) with 95% CIs were reported. Kaplan–Meier curves were generated to estimate glaucoma risk across tertiles of cumulative dose for each glaucoma subtype, and differences between tertile groups were assessed using the log-rank test. Statistical analyses were conducted using SAS Enterprise Guide (version 7.1; SAS Institute Inc., Cary, NC, United States) and R software (version 4.0.3; R Foundation for Statistical Computing, Vienna, Austria). All *P* values were two-sided, and no adjustments were made for multiple comparisons.

## 3 Results

### 3.1 Food and Drug Administration Adverse Event Reporting System analysis

Table 1 summarizes the glaucoma-related adverse events reported for diuretics in the FAERS database up to December 2024. The table shows a wide range of RORs for different diuretics. Hydrochlorothiazide, chlorthalidone, and furosemide were the three diuretics with the highest number of glaucoma reports. Among these, hydrochlorothiazide and chlorthalidone exhibited pronounced disproportionality signals, with RORs exceeding 2 (3.34; 95% CI, 2.85–3.91 and 11.37; 95% CI, 7.67–16.86, respectively), IC025 values above 0 (1.48 and 2.36, respectively), EBGM05 values above 2.0 (2.91 and 8.13, respectively), and number of reports exceeding 10 (155 and 25, respectively). Supplementary Table S3 presents the glaucoma-related adverse reactions reported for diuretics, including the number of reports and their relative frequencies in the FAERS database. Notably, chlorthalidone and furosemide showed a higher frequency of adverse events coded as angle-closure glaucoma than other glaucoma subtypes.

### 3.2 Korean Health Insurance Review and Assessment Service database analysis

Supplementary Table S4 summarizes the clinical characteristics and prescription details of patients in the population-based HIRA cohort for hydrochlorothiazide and chlorthalidone. The mean age was comparable between groups: 60.3 ± 15.6 years for hydrochlorothiazide users and 59.4 ± 15.1 years for chlorthalidone users. Males comprised 52.2% of hydrochlorothiazide users and 59.8% of chlorthalidone users, respectively. The mean duration of drug exposure was 6.6 ± 6.8 months in the hydrochlorothiazide cohort and 6.9 ± 6.7 months in the chlorthalidone cohort.

Table 2 presents the incidence rates and corresponding IRRs for each glaucoma type before and after initiation of the two drugs. The IRR for overall glaucomatous conditions was 1.02 (95% CI, 0.98–1.06) for hydrochlorothiazide and 1.03 (95% CI, 0.84–1.26) for chlorthalidone, indicating no significant increase in glaucoma incidence following drug exposure. A similar pattern was observed for specific subtypes, including actual glaucoma (1.03; 95% CI, 0.94–1.13 for hydrochlorothiazide and 0.97; 95% CI, 0.62–1.51 for chlorthalidone), angle-closure glaucoma (1.06; 95% CI, 0.70–1.62 for hydrochlorothiazide and 0.26; 95% CI, 0.03–2.50 for chlorthalidone), open-angle glaucoma (0.99; 95% CI, 0.88–1.11 and 1.12; 95% CI, 0.64–1.96, respectively), and ocular hypertension (1.02; 95% CI, 0.97–1.07 and 1.09; 95% CI, 0.87–1.37, respectively), none of which showed a statistically significant increase in risk after drug exposure.

**TABLE 2:** Incidence rate ratios of glaucoma before and after drug use.

​	Hydrochlorothiazide		Chlorthalidone	
	Pre-exposure^a^	Post-exposure^b^	Pre-exposure^a^	Post-exposure^b^
Observation period, years, mean (SD)	0.96 (0.58)	1.04 (0.58)	1.03 (0.58)	0.97 (0.58)
Overall glaucomatous conditions				
Events	5,348	3,884	265	152
Rates	1.78	1.81	1.70	1.76
IRR (95% CI)	1.02 (0.98–1.06)		1.03 (0.84–1.26)	
Actual glaucoma				
Events	990	931	41	37
Rates	1.66	1.72	1.96	1.89
IRR (95% CI)	1.03 (0.94–1.13)		0.97 (0.62–1.51)	
Angle-closure glaucoma				
Events	40	47	3	1
Rates	1.51	1.61	2.27	0.58
IRR (95% CI)	1.06 (0.70–1.62)		0.26 (0.03–2.50)	
Open-angle glaucoma				
Events	557	541	25	24
Rates	1.67	1.65	1.83	2.05
IRR (95% CI)	0.99 (0.88–1.11)		1.12 (0.64–1.96)	
Ocular hypertension				
Events	4,383	3,149	222	113
Rates	1.79	1.83	1.67	1.82
IRR (95% CI)	1.02 (0.97–1.07)		1.09 (0.87–1.37)	

^a^ Pre-exposure period = 1 January 2019, to the date of the first prescription.

^b^ Post-exposure period = From the date of first prescription to 31 December 2020.

SD, standard deviation; IRR, incidence rate ratios; CI, confidence interval.

**FIGURE 2:**
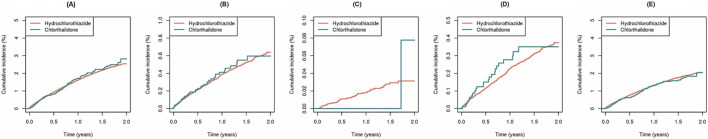
Cumulative incidences of glaucoma subtypes for hydrochlorothiazide and chlorthalidone. Cumulative incidences of **(A)** overall glaucomatous conditions **(B)** actual glaucoma **(C)** angle-closure glaucoma **(D)** open-angle glaucoma, and **(E)** ocular hypertension among hydrochlorothiazide and chlorthalidone users over the study period, estimated using Kaplan–Meier curves. The red lines indicate hydrochlorothiazide users, and the green line represents chlorthalidone users.

Table 3 presents the cumulative and age- and sex-adjusted incidences of glaucoma at three time points: 6 months, 1 year after drug initiation, and study completion. For overall glaucomatous conditions, hydrochlorothiazide demonstrated incidences of 1.0% (age- and sex-adjusted, 0.9%) at 6 months, 1.7% (1.6%) at 1 year, and 2.6% (2.5%) by study completion, with cases of ocular hypertension accounting for the majority of events. Chlorthalidone exhibited comparable findings, with incidences of 0.9% (0.7%) at 6 months, 1.7% (1.5%) at 1 year, and 2.9% (2.5%) by study completion, also primarily driven by ocular hypertension. Figure 2 depicts the cumulative incidence of overall glaucomatous conditions and its subtypes throughout the study period for hydrochlorothiazide and chlorthalidone, demonstrating a gradual increase over time.

**TABLE 3:** Cumulative and age- and sex-adjusted incidences of glaucoma per person-year.

Adverse event	Hydrochlorothiazide, % (age- and sex-adjusted %)			Chlorthalidone, % (age- and sex-adjusted %)		
	6 months	1 year	Up to the study end	6 months	1 year	Up to the study end
Overall glaucomatous conditions	1.0% (0.9%)	1.7% (1.6%)	2.6% (2.5%)	0.9% (0.7%)	1.7% (1.5%)	2.9% (2.5%)
Actual glaucoma	0.2% (0.2%)	0.4% (0.4%)	0.6% (0.7%)	0.2% (0.2%)	0.4% (0.4%)	0.6% (0.6%)
Angle-closure glaucoma	0.01% (0.01%)	0.02% (0.01%)	0.03% (0.02%)	0.00% (0.00%)	0.00% (0.00%)	0.08% (0.11%)
Open-angle glaucoma	0.1% (0.1%)	0.2% (0.3%)	0.4% (0.4%)	0.2% (0.2%)	0.3% (0.3%)	0.4% (0.4%)
Ocular hypertension	0.8% (0.7%)	1.3% (1.3%)	2.1% (1.9%)	0.6% (0.5%)	1.3% (1.1%)	2.1% (1.7%)

Table 4 summarizes the results of the Cox proportional hazards analysis evaluating factors potentially associated with increased glaucoma risk, including drug exposure and duration of use. Hydrochlorothiazide, compared with chlorthalidone as the reference in the active-comparator analysis, was not associated with an increased or decreased risk of overall glaucomatous conditions (HR 1.01; 95% CI, 0.86–1.19) or any glaucoma subtype. Additionally, neither the duration of use nor the daily dose of each drug was consistently associated with an elevated risk across glaucoma outcomes; although longer duration showed a modest association with overall glaucomatous conditions and ocular hypertension (both HR 1.01; 95% CI, 1.01–1.02), it was not significant for other subtypes, and daily dose was not significant for any subtype.

**TABLE 4:** Multivariate Cox proportional hazards models for glaucoma.

Variable	Overall glaucomatous conditions, HR (95% CI)	Actual glaucoma HR (95% CI)	Angle-closure glaucoma, HR (95% CI)	Open-angle glaucoma, HR (95% CI)	Ocular hypertension HR (95% CI)
Hydrochlorothiazide vs. Chlorthalidone^a^	1.01 (0.86–1.19)	1.02 (0.73–1.42)	0.56 (0.08–4.07)	1.13 (0.75–1.70)	0.93 (0.77–1.12)
Duration of use, months	1.01 (1.01–1.02)	1.01 (1.00–1.02)	1.02 (0.98–1.06)	1.01 (0.99–1.02)	1.01 (1.01–1.02)
Daily dose, mg	1.00 (0.99–1.00)	1.00 (0.99–1.01)	1.02 (0.99–1.06)	1.00 (0.99–1.01)	1.00 (0.99–1.00)
Age	1.01 (1.01–1.01)	1.02 (1.01–1.02)	1.03 (1.01–1.06)	1.02 (1.01–1.03)	1.01 (1.00–1.01)
Sex, Female^b^	1.13 (1.06–1.21)	0.87 (0.76–1.00)	1.84 (0.98–3.47)	0.78 (0.66–0.93)	1.19 (1.11–1.28)
Diabetes mellitus	1.36 (1.27–1.45)	1.42 (1.23–1.63)	1.64 (0.89–2.99)	1.48 (1.24–1.77)	1.40 (1.30–1.51)
Hypertension	0.93 (0.82–1.04)	1.19 (0.91–1.55)	2.26 (0.50–10.25)	1.00 (0.72–1.38)	0.86 (0.76–0.98)
Hyperlipidemia	1.27 (1.19–1.36)	1.25 (1.09–1.43)	1.08 (0.59–1.97)	1.31 (1.09–1.56)	1.29 (1.20–1.39)
Kidney disease	1.25 (1.06–1.47)	1.59 (1.19–2.12)	0.48 (0.07–3.55)	1.26 (0.84–1.89)	1.17 (0.97–1.42)
Liver disease	1.13 (1.06–1.21)	1.10 (0.95–1.26)	2.17 (1.18–4.00)	1.02 (0.85–1.23)	1.15 (1.07–1.24)

HR, hazard ratio; CI, confidence interval; mg, milligram.

^a^ Hydrochlorothiazide was used as a reference.

^b^ Male as reference.

### 3.3 Dose–response relationship

**FIGURE 3:**
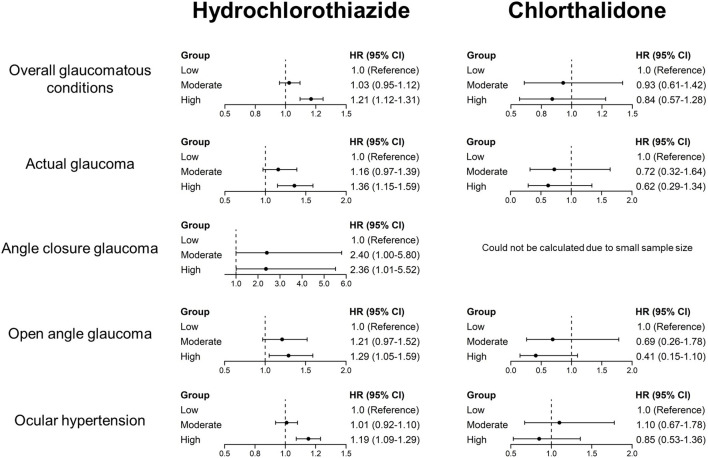
Dose–response relationship between drug exposure and glaucoma, stratified by exposure group (low, moderate, and high exposure) according to the cumulative dose.

Figure 3, Supplementary Figures S1, S2 illustrate the dose–response associations between glaucoma risk and cumulative drug exposure, stratified by tertile dose groups. Figure 3 presents forest plots of hazard ratios for glaucoma outcomes across tertiles of cumulative exposure. In these analyses, higher cumulative exposure to hydrochlorothiazide was associated with glaucoma outcomes, primarily in the highest exposure category, including overall glaucomatous conditions (HR 1.21; 95% CI, 1.12–1.31), actual glaucoma (HR 1.36; 95% CI, 1.15–1.59), angle-closure glaucoma (HR 2.36; 95% CI, 1.01–5.52), open-angle glaucoma (HR 1.29; 95% CI, 1.05–1.59), and ocular hypertension (HR 1.19; 95% CI, 1.09–1.29). In contrast, chlorthalidone showed no significant increase in risk for any glaucoma subtype across cumulative exposure categories (all 95% CIs included 1.00).

Supplementary Figures S1, S2 present Kaplan-Meier analyses of glaucoma incidence stratified by tertiles of cumulative drug exposure, with between-group differences assessed using the log-rank test. For hydrochlorothiazide, cumulative incidence differed significantly across tertiles for overall glaucomatous conditions (P < 0.001), actual glaucoma (P = 0.001), and ocular hypertension (P < 0.001), but not for angle-closure glaucoma (P = 0.092) or open-angle glaucoma (P = 0.057) (Supplementary Figure S1). For chlorthalidone, no significant differences were observed across tertiles for any glaucoma subtype (all P > 0.05) (Supplementary Figure S2).

## 4 Discussion

Using nationwide, real-world data, this study found no significant increase in glaucoma risk associated with the use of two commonly prescribed thiazide diuretics, hydrochlorothiazide and chlorthalidone, despite positive pharmacovigilance signals. Across all analytical models—including IRRs, Cox proportional hazards models, and dose–response analyses—both agents exhibited comparable risks before and after drug exposure, with a modest dose-stratified signal observed only for hydrochlorothiazide at the highest cumulative exposure. These findings underscore the importance of validating drug–adverse reaction associations identified through pharmacovigilance systems using real-world population-based data.

We implemented a two-step approach to identify underrecognized systemic drugs associated with maculopathy: hypothesis generation (screening) using FAERS and validation using the HIRA database, paralleling our methodology previously applied to maculopathy (Kim et al., 2025a). While previous FAERS disproportionality analyses reported several drugs potentially associated with glaucoma (Aftab et al., 2024; Wu et al., 2025), real-world validation remained limited. Case studies have described angle-closure glaucoma associated with hydrochlorothiazide and chlorthalidone use (Geanon and Perkins, 1995; Lee et al., 2007; Chen et al., 2014; Singer et al., 2015; Durai et al., 2016; Wu et al., 2023; Premkumar et al., 2025), and a DIN-LINK (UK) case–control analysis (Owen et al., 2010), along with analyses of FAERS data (Aftab et al., 2024; Wu et al., 2025), have also identified these drugs as potential glaucoma-inducing agents. Given that early recognition and prevention are critical in drug-induced glaucoma (Wu et al., 2025), clinicians should recognize high-risk drugs and exercise caution in susceptible patients (Razeghinejad et al., 2011), without unwarranted discontinuation of essential medications. Validating FAERS signals with real-world data helps confirm true drug–ocular adverse reaction associations and prevents unnecessary medication withdrawal.

Hydrochlorothiazide and chlorthalidone, both sulfonamide derivatives, are widely prescribed as thiazide and thiazide-like antihypertensive diuretics. Although rare, hydrochlorothiazide (Geanon and Perkins, 1995; Soylev et al., 1995; Lee et al., 2007; Roh et al., 2011; Chen et al., 2014; Wu et al., 2023; Premkumar et al., 2025) and chlorthalidone (D'Alena and Robinson, 1969; Mahesh et al., 2007; Singer et al., 2015; Durai et al., 2016) can precipitate idiosyncratic uveal (ciliochoroidal) effusions that rotate the ciliary body anteriorly and displace the iris–lens diaphragm forward, resulting in anterior chamber shallowing (D'Alena and Robinson, 1969; Geanon and Perkins, 1995; Soylev et al., 1995; Lee et al., 2007; Roh et al., 2011; Chen et al., 2014; Wu et al., 2023; Premkumar et al., 2025), an acute myopic shift (D'Alena and Robinson, 1969; Geanon and Perkins, 1995; Soylev et al., 1995; Mahesh et al., 2007; Roh et al., 2011; Premkumar et al., 2025), and non–pupillary-block secondary angle closure (Geanon and Perkins, 1995; Lee et al., 2007; Chen et al., 2014; Singer et al., 2015; Durai et al., 2016; Wu et al., 2023; Premkumar et al., 2025). However, in this study, we found no association between the use of hydrochlorothiazide or chlorthalidone and glaucoma, suggesting that while uveal effusion is a plausible mechanism for drug-induced angle closure in individual cases, it does not translate into an increased glaucoma risk at the population level.

In a recent FAERS-based study evaluating drugs associated with increased glaucoma risk, hydrochlorothiazide was classified as a medium-risk glaucoma-inducing drug (Wu et al., 2025), and both hydrochlorothiazide and chlorthalidone demonstrated positive safety signals for angle-closure glaucoma (Aftab et al., 2024). However, neither agent showed a significant association with acute angle-closure glaucoma in a case-crossover health-claims study (Na and Park, 2022) or in a case–control study using the PharMetrics Plus database (IQVIA, United States) (Qiao et al., 2024). Regarding elevated IOP or overall glaucomatous conditions, the Gutenberg Health Study (Laspas et al., 2024) reported that continuous, discontinued, or new thiazide use was not associated with IOP changes over a 5-year follow-up period. Similarly, the Rotterdam Study (Muskens et al., 2007) and a meta-analysis of 11 population-based European cohorts (European Eye Epidemiology Consortium) (Vergroesen et al., 2023) demonstrated that low-ceiling diuretics, including thiazides or thiazide-like diuretics, were not associated with incident open-angle glaucoma or IOP changes. The absence of associations in real-world data, including in our study, may reflect the extremely low incidence of hydrochlorothiazide- or chlorthalidone-induced glaucoma or potential misclassification of angle-closure cases. To minimize potential misclassification bias, we examined multiple outcomes, including overall glaucomatous conditions (all glaucoma-related diagnostic codes, excluding secondary glaucoma of other etiologies) and specific subtype definitions. Nevertheless, our claims-based analyses did not identify a significant association between these thiazide diuretics and any glaucoma outcomes.

Differences between the FAERS and HIRA findings likely reflect both dataset characteristics and the rarity of the events of interest. Regarding dataset characteristics, the FAERS and HIRA databases represent fundamentally different structures of healthcare big data. FAERS is a spontaneous reporting system that serves as an early warning tool for potential safety signals (Fang et al., 2014; Jiang et al., 2025), but it cannot determine incidence or establish causality (Chedid et al., 2018; Lucas et al., 2022). Because multiple drugs are frequently reported within a single FAERS case, confounding by co-reporting may produce spurious associations (Food and Drug Administration, 2023), and prior reports suggest that glaucoma risk may not increase with diuretics alone but may rise when combined with other agents (Lee et al., 2023a); therefore, confounding by concomitant therapy cannot be excluded. Moreover, the lack of detailed temporal information on drug exposure and adverse events further limits causal inference (Jiang et al., 2025). In addition, case reports and spontaneous reporting–based disproportionality analyses are prone to stimulated reporting and selective over-reporting of rare or unexpected adverse events; thus, FAERS signals may reflect reporting behavior or hypothesis-generating associations rather than true population-level risk. In contrast, HIRA provides longitudinal, population-based claims data that more comprehensively capture clinically diagnosed glaucoma events and enable estimation of incidence rates, supporting real-world validation of FAERS signals (Kim et al., 2014; Kim et al., 2017; Na and Park, 2022; Kim et al., 2023; Kim et al., 2024; Kwon et al., 2024). HIRA incorporates prescription timing, exposure duration, and diagnostic confirmation, enabling more accurate and temporally defined risk estimation (Kim et al., 2017). These distinctions likely account for the lack of concordance between pharmacovigilance signals from FAERS and population-based outcomes from HIRA, underscoring the importance of integrating both data sources to achieve a balanced and accurate evaluation of drug-related glaucoma.

Furthermore, thiazide-induced angle-closure events may generate disproportionate pharmacovigilance signals in FAERS yet remain statistically non-significant in HIRA analyses when the events are extremely rare. Even for topiramate, a sulfonamide derivative associated with one of the highest glaucoma RORs reported in FAERS (Aftab et al., 2024; Wu et al., 2025), the population-based incidence of ciliochoroidal effusion is extremely low (approximately 3 per 100,000 users) (Abtahi et al., 2012). Given that thiazide diuretics produce substantially lower disproportionality signals (Aftab et al., 2024; Wu et al., 2025), their true incidence is likely very low, although direct comparisons between FAERS disproportionality metrics and population-based incidence should be interpreted cautiously. Moreover, mild or self-resolving effusions may not progress to elevated IOP or angle closure (Soylev et al., 1995; Roh et al., 2011) and, therefore, may remain undetected in claims-based datasets. Despite employing pre- and post-exposure comparative analyses to enhance detection sensitivity, the extreme rarity of such events inherently limits statistical power, highlighting why pharmacovigilance signals may not always translate into confirmatory population-based evidence.

Additionally, population heterogeneity may have contributed to discrepancies between FAERS signals and HIRA outcomes. FAERS aggregates spontaneous reports from heterogeneous populations across multiple countries, whereas HIRA represents a more homogeneous, single-nation cohort. Such differences in genetic backgrounds, comorbidities, prescribing behaviors, and healthcare systems may further influence observed effect estimates. However, previous reports using the PharMetrics Plus database (IQVIA, United States) (Qiao et al., 2024) and European cohorts (Muskens et al., 2007; Vergroesen et al., 2023; Laspas et al., 2024) have reported findings consistent with ours, indicating that population heterogeneity alone is unlikely to explain the observed discrepancies between FAERS and HIRA.

Notably, dose-stratified analyses provided hints of some dose–response patterns, with elevated HRs in the highest cumulative hydrochlorothiazide exposure group for overall glaucomatous conditions, actual glaucoma, and ocular hypertension. These findings should be interpreted cautiously because they may reflect chance variation due to multiple comparisons or residual confounding, as patients receiving higher cumulative doses may differ systematically in age, comorbidity burden, or healthcare utilization despite multivariable adjustment. Therefore, future studies with longer follow-up and more careful adjustment for characteristics associated with high exposure are warranted to more definitively evaluate potential dose-dependent risks.

Our study has the following clinical implications. While isolated case reports document idiosyncratic reactions to thiazide diuretics (e.g., ciliochoroidal effusion precipitating acute secondary angle closure), our nationwide cohort analysis provides reassurance that hydrochlorothiazide and chlorthalidone do not appear to substantially increase the risk of glaucoma at the population level. Although clinicians should consider the possibility of glaucoma-related symptoms in patients treated with these agents, such concerns should not exclude their use or justify discontinuation of effective antihypertensive therapy in the absence of clinical signs.

This study has several limitations. First, FAERS data were derived from spontaneous, voluntary reports, which are subject to underreporting and potential bias influenced by external factors such as publications or media coverage (Jiang et al., 2025). Second, in the HIRA dataset, glaucoma diagnoses were identified using claims codes rather than medical records, which may limit diagnostic accuracy and prevent verification of actual drug adherence or clinical context (Kim et al., 2017). To address limitations of claims-based outcome definitions, we considered incorporating glaucoma medication prescriptions to improve case ascertainment; however, this was not feasible in our study due to medication data limitations (code overlap for some glaucoma medication with other codes in HIRA). Therefore, we mitigated misclassification by requiring at least two diagnosis codes recorded by ophthalmologists. Discordance in glaucoma terminology between HIRA and FAERS may be a potential source of bias. Specifically, FAERS accepts patient reports and uses broad, nonspecific categories such as “glaucoma,” making it difficult to subdivide conditions using the International Classification of Diseases, 10th Revision codes, as in HIRA. Therefore, we used multiple glaucoma-related terms solely to identify potential glaucomatous adverse reactions, rather than detailed analyses of specific subtypes. Notably, elevated IOP in HIRA (H40.0) is not coded exclusively as ocular hypertension; coding can overlap with glaucoma suspect, limiting differentiation based on claims data alone. Moreover, because the HIRA cohort represents a relatively homogeneous Korean population, the generalizability of these findings to other populations may be limited. Additionally, isolating the effect of diuretics from the influence of underlying diseases is also challenging, raising concerns about confounding by indication and disease severity. For instance, cardiovascular comorbidities and uncontrolled hypertension are established risk factors for glaucoma (Lee et al., 2023b), and diuretic use may indicate the presence of these conditions. Concomitant use of non-thiazide diuretics (e.g., loop or potassium-sparing agents) was permitted and may have introduced residual confounding; however, these classes did not exhibit positive signals in our FAERS analyses. Furthermore, regarding the study design, self-controlled pre–post comparisons may be vulnerable to detection bias and time-varying confounding, as healthcare utilization and ophthalmologic evaluations often increase after medication initiation, potentially inflating post-exposure diagnoses of elevated IOP or glaucoma independent of a causal effect. Nevertheless, this design was selected to control for fixed individual-level characteristics (e.g., genetics and baseline health status), particularly in the absence of an appropriate active comparator group. Of note, despite this potential bias toward higher post-exposure detection, no significant increase in incidence was observed after drug initiation. In addition, monitoring for glaucoma outcomes continued regardless of treatment discontinuation because glaucomatous damage may occur or be diagnosed after cessation of therapy; however, this intention-to-treat–style approach may bias effect estimates if the true risk is confined to periods of active use. A longer cohort accrual or follow-up period could have yielded more glaucoma events and increased statistical power. Nonetheless, prior evidence indicates that any excess glaucoma risk associated with diuretics is concentrated early, mostly within the first 2 years (Owen et al., 2010; Lee et al., 2023a). These patterns suggest that a 2-year observation window is sufficient to capture the clinically relevant association between diuretic exposure and glaucoma risk, although longer follow-up could improve precision. Further validation using larger, ethnically diverse, and independently curated databases is warranted to strengthen the robustness of these findings.

In summary, we applied a two-step methodology combining FAERS and HIRA data to identify and evaluate diuretics associated with glaucoma, providing complementary insights into drug-related glaucoma risk. FAERS analyses serve a hypothesis-generating role, detecting preliminary safety signals for the two commonly used thiazide diuretics. Meanwhile, HIRA offers population-based, real-world validation, enabling stronger causal inference and the assessment of dose–response relationships. Together, these complementary data sources strengthen the interpretation of pharmacovigilance and pharmacoepidemiologic findings. Based on our findings, thiazide diuretics should not be avoided solely due to the potential risk of glaucoma; however, future investigations are warranted to assess this association in larger populations and over the long term.

## Data Availability

The raw data supporting the conclusions of this article will be made available by the authors, without undue reservation.

## Supplementary material

SUPPLEMENTARY FIGURE S1 Kaplan–Meier curves by cumulative hydrochlorothiazide exposure tertiles. Kaplan–Meier estimates of cumulative incidence (%) for glaucoma outcomes stratified by tertiles of cumulative hydrochlorothiazide exposure (low, moderate, and high) from the index date. Panels show **(A)** overall glaucomatous conditions, **(B)** actual glaucoma, **(C)** angle-closure glaucoma, **(D)** open-angle glaucoma, and **(E)** ocular hypertension. Between-tertile differences were assessed using the log-rank test; P values are shown in each panel.

SUPPLEMENTARY FIGURE S2 Kaplan–Meier curves by cumulative chlorthalidone exposure tertiles. Kaplan–Meier estimates of cumulative incidence (%) for glaucoma outcomes stratified by tertiles of cumulative chlorthalidone exposure (low, moderate, and high) from the index date. Panels show **(A)** overall glaucomatous conditions, **(B)** actual glaucoma, **(C)** angle-closure glaucoma, **(D)** open-angle glaucoma, and **(E)** ocular hypertension. Between-tertile differences were assessed using the log-rank test; P values are shown in each panel.
