# Supplementary figures and images for: Hydrochlorothiazide and chlorthalidone use and glaucoma risk: pharmacovigilance analysis and nationwide cohort study
Source: Front Pharmacol. 2026 Mar 10;17:1768133. doi: 10.3389/fphar.2026.1768133 (PMC13008922; doi:10.3389/fphar.2026.1768133)

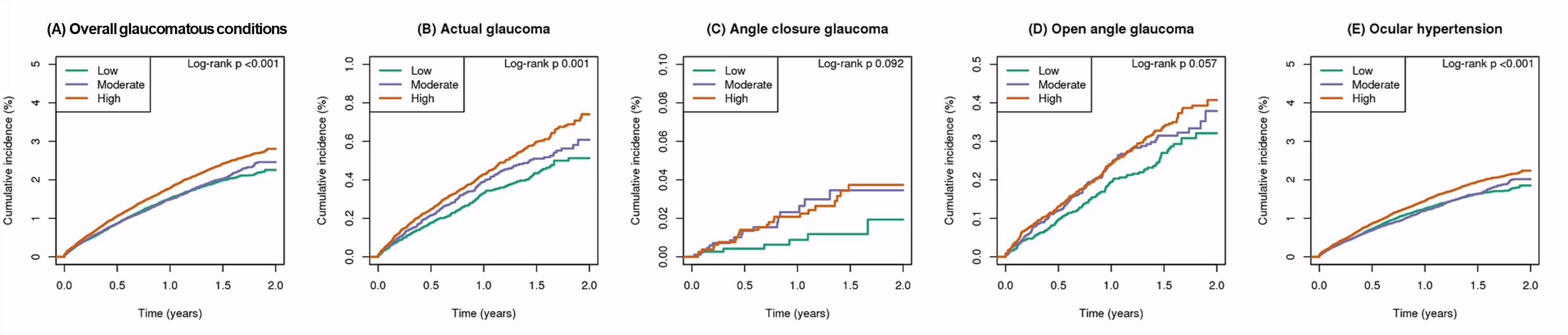

Supplement: Supplementary file 1 [file Image1.tiff]

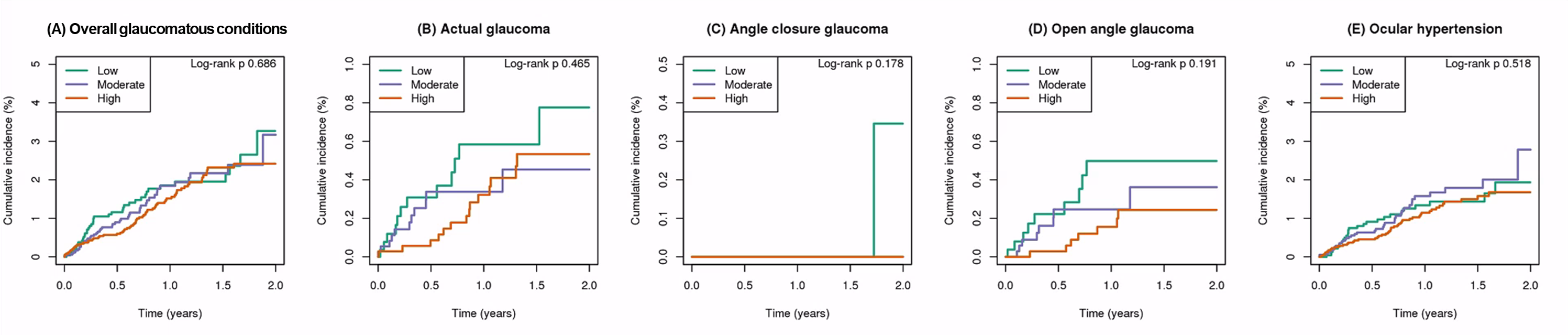

Supplement: Supplementary file 6 [file Image2.tiff]
