# Supplementary material for: Hydrochlorothiazide and chlorthalidone use and glaucoma risk: pharmacovigilance analysis and nationwide cohort study
Source: Front Pharmacol. 2026 Mar 10;17:1768133. doi: 10.3389/fphar.2026.1768133 (PMC13008922; doi:10.3389/fphar.2026.1768133)
Supplement: Supplementary file 2 [file Table1.docx]

**Supplementary Table S1.** Food and Drug Administration Adverse Event Reporting System (FAERS) terms used to identify glaucoma-related adverse events

| **FAERS terms** | **Category** |
| --- | --- |
| Intraocular Pressure Increased | Ocular hypertension |
| Glaucoma | Glaucoma |
| Glaucomatous Optic Neuropathy |  |
| Malignant glaucoma |  |
| Angle-closure Glaucoma | Angle-closure glaucoma |
| Open-angle glaucoma | Open angle glaucoma |
| Normal tension glaucoma |  |
