# Supplementary material for: Hydrochlorothiazide and chlorthalidone use and glaucoma risk: pharmacovigilance analysis and nationwide cohort study
Source: Front Pharmacol. 2026 Mar 10;17:1768133. doi: 10.3389/fphar.2026.1768133 (PMC13008922; doi:10.3389/fphar.2026.1768133)
Supplement: Supplementary file 3 [file Table2.docx]

**Supplementary Table S2.** Definitions of Korean Classification of Diseases (KCD) codes (adapted from the International Statistical Classification of Diseases and Related Health Problems, 10th Revision [ICD-10]) used to identify glaucoma

| **KCD codes** | **KCD terms** | **Category** | | |
| --- | --- | --- | --- | --- |
| H40.0 | Glaucoma suspect/ocular hypertension | Ocular hypertension | | Overall glaucomatous conditions |
| H40.1 | Primary open-angle glaucoma | Open-angle glaucoma | Actual glaucoma |  |
| H40.2 | Primary angle-closure glaucoma | Angle-closure glaucoma |  |  |
| H40.6 | Glaucoma secondary to drugs | Other glaucoma |  |  |
| H40.8 | Other glaucoma |  |  |  |
| H40.9 | Unspecified glaucoma |  |  |  |

* Glaucoma diagnosis required ≥2 outpatient claims bearing the same glaucoma subtype diagnosis code, recorded by an ophthalmologist.
