# Supplementary material for: Hydrochlorothiazide and chlorthalidone use and glaucoma risk: pharmacovigilance analysis and nationwide cohort study
Source: Front Pharmacol. 2026 Mar 10;17:1768133. doi: 10.3389/fphar.2026.1768133 (PMC13008922; doi:10.3389/fphar.2026.1768133)
Supplement: Supplementary file 4 [file Table3.docx]

**Supplementary Table S3.** Reported glaucoma-associated adverse reactions among diuretic users and their frequencies in the Food and Drug Administration Adverse Event Reporting System (FAERS) database

| **Drugs** | **Glaucoma** | **Ocular hypertension** | **Angle-closure glaucoma** | **Open-angle glaucoma** |
| --- | --- | --- | --- | --- |
| Hydrochlorothiazide | 88 (56.8%) | 27 (17.4%) | 31 (20%) | 9 (5.8%) |
| Chlorthalidone | 0 | 0 | 25 (100%) | 0 |
| Furosemide | 15 (42.9%) | 1 (2.9%) | 19 (54.3%) | 0 |
| Spironolactone | 9 (69.2%) | 4 (30.8%) | 0 | 0 |
