# Supplementary material for: Hydrochlorothiazide and chlorthalidone use and glaucoma risk: pharmacovigilance analysis and nationwide cohort study
Source: Front Pharmacol. 2026 Mar 10;17:1768133. doi: 10.3389/fphar.2026.1768133 (PMC13008922; doi:10.3389/fphar.2026.1768133)
Supplement: Supplementary file 5 [file Table4.docx]

**Supplementary Table S4.** Clinical characteristics and prescription details of the patients included in the population-based cohort using the Korean Health Insurance Review and Assessment Service (HIRA) database

| **Characteristics** | **Number (%) or mean (SD)** | |
| --- | --- | --- |
|  | **Hydrochlorothiazide**  **(n = 250,296)** | **Chlorthalidone**  **(n = 10,005)** |
| **Age** (SD), years | 60.3 (15.6) | 59.4 (15.1) |
| < 20 | 1323 (0.5%) | 17 (0.2%) |
| 20–39 | 19306 (7.7%) | 920 (9.2%) |
| 40–59 | 105927 (42.3%) | 4256 (42.5%) |
| 60–79 | 89959 (35.9%) | 3714 (37.1%) |
| ≥ 80 | 33781 (13.5%) | 1098 (11.0%) |
| **Sex** |  |  |
| Male | 130680 (52.2%) | 5982 (59.8%) |
| Female | 119636 (47.8%) | 4023 (40.2%) |
| **Medical specialties of the prescriber** |  |  |
| Internal medicine | 168333 (67.3%) | 7799 (78.0%) |
| Orthopedic Surgery | 16747 (6.7%) | 630 (6.3%) |
| Family Medicine | 13890 (5.6%) | 384 (3.8%) |
| **Medical indications for use (multiple diagnoses allowed)** |  |  |
| Essential (primary) hypertension | 203092 (81.1%) | 8396 (83.9%) |
| Disorders of lipoprotein metabolism and other lipidemias | 81843 (32.7%) | 4421 (44.2%) |
| Type 2 diabetes mellitus | 43452 (17.4%) | 1937 (19.4%) |
| **Systemic diseases** |  |  |
| Diabetes mellitus | 80002 (32.0%) | 3623 (36.2%) |
| Hypertension | 214966 (85.9%) | 9066 (90.6%) |
| Dyslipidemia | 84145 (33.6%) | 4036 (40.3%) |
| Kidney disease | 5899 (2.4%) | 461 (4.6%) |
| Liver disease | 82717 (33.1%) | 3737 (37.4%) |
| **Mean daily dose** (SD), mg | 15.9 (7.6) | 16.4 (6.9) |
| **Mean DDD*** (SD), mg | 0.6 (0.3) | 0.7 (0.3) |
| **Mean duration of use** (SD), months | 6.6 (6.8) | 6.9 (6.7) |
| **Mean cumulative dose** (SD), g | 2.8 (3.1) | 3.2 (3.4) |

SD, standard deviation; mg, milligram; DDD, defined daily dose; g, gram.
